# Ethnicity-specific factors influencing childhood immunisation decisions among Black and Asian Minority Ethnic groups in the UK: a systematic review of qualitative research

**DOI:** 10.1136/jech-2016-207366

**Published:** 2017-05-12

**Authors:** Alice S Forster, Lauren Rockliffe, Amanda J Chorley, Laura A V Marlow, Helen Bedford, Samuel G Smith, Jo Waller

**Affiliations:** 1 Health Behaviour Research Centre, UCL, London, UK; 2 Institute of Child Health, UCL, London, UK; 3 Wolfson Institute of Preventive Medicine, Queen Mary University of London, London, UK

**Keywords:** VACCINATION, SYSTEMATIC REVIEWS, ETHNICITY

## Abstract

**Background:**

Uptake of some childhood immunisations in the UK is lower among those from some Black and Asian Minority Ethnic (BAME) backgrounds. This systematic review of qualitative research sought to understand the factors that are associated with ethnicity that influence the immunisation decisions of parents from BAME backgrounds living in the UK.

**Methods:**

Databases were searched on 2 December 2014 for studies published at any time using the terms ‘UK’ and ‘vaccination’ and ‘qualitative methods’ (and variations of these). Included articles comprised participants who were parents from BAME backgrounds. Thematic synthesis methods were used to develop descriptive and higher order themes. Themes specific to ethnicity and associated factors are reported.

**Results:**

Eight papers were included in the review. Most participants were from Black (n=62) or Asian (n=38) backgrounds. Two ethnicity-related factors affected immunisation decisions. First, factors that are related to ethnicity itself (namely religion, upbringing and migration, and language) affected parents' perceived importance of immunisations, whether immunisations were permitted or culturally acceptable and their understanding of immunisation/the immunisation schedule. Second, perceived biological differences affected decision-making and demand for information.

**Conclusions:**

Factors related to ethnicity must be considered when seeking to understand immunisation decisions among parents from BAME backgrounds. Where appropriate and feasible, vaccination information should be targeted to address beliefs about ethnic differences held by some individuals from some BAME backgrounds.

## Introduction

In the UK, the routine childhood programme offers immunisation against 17 diseases, starting when the infant is 2 months old.^1^ Additional vaccines (such as BCG) are offered selectively from birth to high-risk individuals. High uptake is crucial to the success of the programme, providing direct protection to vaccinated individuals and, if a sufficiently high proportion of the population is immunised, indirect protection to the unimmunised through herd immunity. While overall coverage of immunisations in the UK is very good,^2^ ^3^ there is evidence that uptake of some immunisations is lower among individuals from some Black and Asian Minority Ethnic (BAME) backgrounds.^4–12^ While herd immunity will benefit the general population, where subpopulations live apart from the general population they may not be protected if they remain unimmunised. Individuals from some BAME backgrounds live in areas with high concentrations of individuals from their own ethnic group,^13–15^ particularly in cities. Low uptake of immunisations makes individuals living in these ethnically dense areas more vulnerable to disease. In addition, unprotected individuals travelling to countries where infectious diseases remain prevalent may be at risk of acquiring infection and subsequently importing it into the UK, and are vulnerable to infection acquired from unprotected family members visiting the UK from such countries. Although low immunisation uptake is often associated with deprivation,^6^ ^11^ ethnic differences in uptake persist even after controlling for deprivation.^6^ ^7^ ^11^ ^16^ A study of diphtheria immunisation coverage in London found that immunisation uptake varied by ethnicity, but no relationship exists between deprivation and coverage for most ethnic groups (apart from White British and ‘ethnicity not known’ groups),^12^ suggesting that ethnicity is important in understanding uptake of immunisation, independent of deprivation.

Ethnicity was defined by Bhopal in 2004^17^ as a ‘multifaceted quality that refers to the group to which people belong, and/or are perceived to belong, as a result of certain shared characteristics, including geographical and ancestral origins, but particularly cultural traditions and languages’*.* Bhopal states that ethnicity is different from race, nationality, religion and migrant status, but can include facets of these factors. BAME groups in the UK are often considered to be those from non-White British backgrounds. However, ethnic groups are not stable^18^ and change with the social context, and ethnic diversity in the UK is growing.^19^

A number of reviews have provided frameworks to help us understand parents' immunisation decisions,^20–23^ but none has specifically considered parents from BAME backgrounds. In the present study, we systematically reviewed the qualitative literature with the aim of understanding the factors associated with ethnicity that influence the immunisation decisions of parents from BAME backgrounds living in the UK. While we can learn from the primary research studies conducted specifically with individuals from BAME backgrounds, research has also been conducted with general population samples that include individuals from BAME backgrounds but do not comment on ethnicity. The present review allowed an exploration of the role of ethnicity hidden within articles whose focus was not ethnicity, as well as those whose focus was BAME groups. Lower uptake of immunisations among individuals from some minority groups is not a phenomenon limited to the UK.^24–26^ However, we focus solely on the UK for the present review as it is difficult to compare the experiences of BAME groups across countries as their history and migration experiences will vary considerably. Although BAME groups may be defined similarly in different countries, because of different patterns of immigration, they may not have the same composition. The proportion of individuals from different ethnic backgrounds also differs between countries. Appreciation of the factors related to ethnicity that are involved in immunisation decision-making among parents from BAME backgrounds will facilitate the development of interventions that enable parents to make more informed immunisation decisions.

## Methods

A systematic review of qualitative studies was conducted to understand the factors related to ethnicity influencing childhood immunisation decisions of parents from BAME backgrounds living in the UK. The review focused on immunisations offered as part of the UK childhood immunisation programme.^1^ BAME was defined as being not White English/Welsh/Scottish/Northern Irish/British based on 2011 census data which indicated that this is the largest ethnic group in the UK.^27^ ^i^ PsycINFO, MEDLINE, CINAHL Plus, Embase, Social Policy and Practice and Web of Science were searched on 2 December 2014 using the terms ‘vaccination’, ‘qualitative’ and ‘United Kingdom’ (and variants of these terms; see online supplementary material for full search terms). There were no date restrictions. We did not include ‘ethnicity’ as a search term as this was likely to miss articles that included participants from BAME backgrounds, but whose focus was not ethnicity. We included articles that were published in peer-reviewed journals, in English, if they reported qualitative findings (such as those from interviews, focus groups or free-text survey responses) of studies conducted with parents/guardians from BAME backgrounds. Where White British (ie, White English/Welsh/Scottish/Northern Irish/British) and BAME parents were included in studies, we excluded papers where none of the results could be attributed to parents from BAME backgrounds specifically. We excluded letters, dissertations, book chapters, reviews and commentaries. We reviewed reference lists of included articles and conducted forward citation searching using Web of Science.

10.1136/jech-2016-207366.supp1 supplementary material

After combining the results of the six databases, AF removed duplicates and excluded articles if the title obviously did not meet inclusion criteria. Abstracts and then full texts were reviewed by AF/SS/LR/AC, and excluded articles were checked by another researcher. We extracted data on the methods used in the studies and sample characteristics using a piloted Microsoft Excel form. Outcome data were participant quotes or authors' interpretation of qualitative data that had been reported in the Results section of the article or abstract (imported into NVIVO; QSR International Pty: NVIVO qualitative data software, 10 edn; 2012).

We used thematic synthesis to analyse the data, following the methods described by Thomas and Harden.^28^ The aim of thematic synthesis is to generate new knowledge or conceptual innovations and provide findings that go beyond those of the primary studies. Thematic analysis is a qualitative analytical method and borrows techniques from quantitative systematic review methods, affording a high level of rigour. First, text was coded line by line (or by thematic fragment) by AF, AC and LR (one-third each) and descriptive themes developed through lengthy discussion conducted over three sessions. Analytical themes were then generated by discussion with AF, AC, LR, LM and JW until consensus on interpretation was reached. We used NVivo to code and group data into themes (QSR International Pty, 2012). We report findings that related specifically to ethnicity or associated factors (such as religion). Issues unrelated to ethnicity were raised in the data but are not discussed in the results (see online supplementary material). We assessed study quality using the Critical Appraisal Skills Programme (CASP) tool.^29^ Studies with scores of 0–4 had a high risk of bias, and 5–9 low risk. Findings are reported in line with Preferred Reporting Items for Systematic Reviews and Meta-Analyses (PRISMA) guidelines.^30^ Quotes are presented detailing the lead author of the article and whether the quote came from a participant or author.

## Results

### Summary of included articles

**Figure 1:**
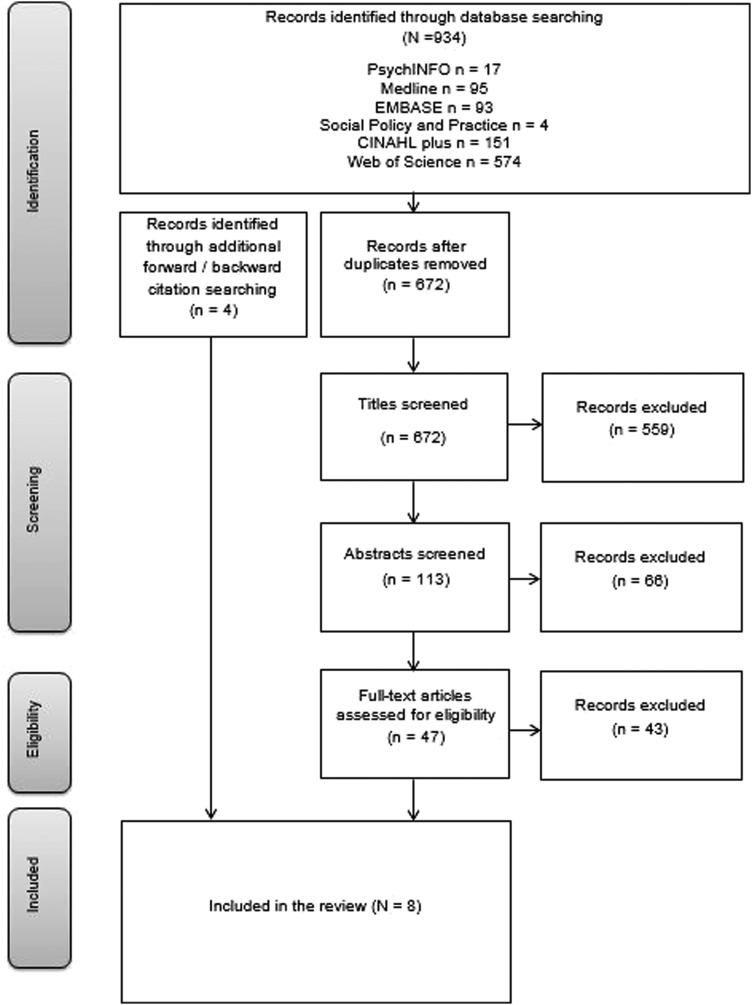
Flow diagram of included studies, adapted from Moher *et al*.^30^

The search identified 934 articles (see figure 1 for flow of inclusion/exclusion). In total, eight articles were included comprising a total of 209 participants from BAME backgrounds (range: 1–64; see online supplementary material for table of included studies and full references). One article included a specific BAME group, two included a BAME sample in general and five included BAME participants in a general population sample. Articles used interviews (n=5) and/or focus groups (n=4). Data were most commonly analysed using thematic analysis (n=3; framework analysis=2, grounded theory=2, analytic deduction=1). All articles were rated as having low risk of bias. Five articles focused on measles, mumps and rubella (MMR) immunisation, two on immunisations in general and one on human papillomavirus (HPV).

There were 61 participants in the studies from Black/African/Caribbean/Black British backgrounds, 37 were from Asian/Asian British backgrounds and 6 were White non-British (other ethnicities included Chinese=1, Turkish=1, mixed ethnicity=1, Eastern European=1^ii^). Only three studies explicitly reported that participants self-reported their ethnicity. One article reported ethnic group and religion and included seven Black Christians, five Asian Muslims, four Asian Hindus, two Black participants with no religion, one Asian Christian and one Black Muslim. Participants' religions in others studies included Islam, Hinduism and Christianity. Where migration data were available, 12 participants were born in the UK and 31 were not. The languages spoken included English, Somali, Punjabi, Urdu and Gujarati. In two studies, data were collected in English only, in four studies data were collected in English and other languages and two studies did not specify. Most participants were mothers.

### Summary of findings

Ethnicity affected immunisation decisions in two ways. First, factors related to ethnicity influenced parents' perceptions about immunisations and second, beliefs about biological ethnic differences altered parents' perceived susceptibility to disease and vaccine side effects.

#### Factors related to ethnicity influence perceptions about immunisations

##### Religion

Parents' religion affected their perceptions of the importance of immunisations and whether immunisations were permitted. Some mothers stated that their antipreventive medicine beliefs were linked to their religion. Contrarily, some Somali, Hindu and Asian Hindu mothers described how their religion taught them to look after the health of their children and so influenced their decision to vaccinate.

I do believe it [immunisations]… reduce diseases. But God knows—he can bring the kids to be sick or not to be sick basically. Tomlinson, participant comment.

…In our religion it says whatever that's good for your health…just do it. Tomlinson, participant comment.

Religious practices also affected whether parents believed their children were at risk of acquiring immunisation-preventable diseases. Some Asian Muslim and Black Christian parents discussing HPV immunisation (which protects against a sexually transmitted infection) stated that their daughters were not at risk of infection, as their religion promotes abstinence from sex before marriage. However, other Asian Muslim parents opted to vaccinate against HPV as they believed that they could not control their children's behaviour.

Coming from a Muslim background… we don't have sex before marriage for example, so your first experiences of these things are when you're married and you stay in a relationship… because of that reason I'd probably say no, I wouldn't bother with it with my two girls. Marlow, participant comment.

Some Somali parents had concerns about the content of immunisations (gelatine) on religious grounds.

##### Upbringing and migration

Parents' upbringing played a role in their decision-making. Black Muslim and Black Christian mothers explained that their positive attitudes towards immunisation were a result of knowing about poor health in other countries. In addition, these mothers' experience of healthcare in other countries made them appreciate that free healthcare is not assured. Similarly, one Somali mother was influenced by the immunisation practices in her ‘home’ country and another described how conflict in her country of birth meant she did not have knowledge about immunisation. Some Somali parents' lack of exposure to immunisation-preventable diseases ‘back home’ reduced the perceived importance of unfamiliar immunisations.

One mother believed that the immunisations against tuberculosis is the most important immunisation, as ‘that's the only important one that they do back home'. Tomlinson, author and participant comment.

In one study, parents who had not grown up in the UK had inaccurate beliefs about the UK healthcare system. Some Somali mothers expressed concern that their child would not be able to go to school without completing their childhood immunisations or there would be social consequences, but accepted this as part of the ‘*British system’*.

##### Language

Language issues made it difficult for some parents to get their children vaccinated. Parents who did not speak English were concerned their children would not get the right immunisation or would get an immunisation twice. Parents wanted information to be available in their mother tongue, although one article reported that participants also had difficulty reading information in their mother tongue. In one article, parents who were unable to read English were shielded from adverse coverage of immunisations, as were parents who read Indian newspapers, which did not have such coverage and resulted in parents having fewer immunisation concerns.

…the Gujarati group was unable to read English, and the Indian newspapers had little coverage of the MMR vaccination debate in the UK. Mixer, author comment.

#### Beliefs about biological ethnic differences

Some parents' beliefs about immunisation were influenced by their beliefs about biological differences between themselves and the majority population in the UK or belief that the UK environment is different from their country of birth. These beliefs affected vaccination decisions in a number of ways and the information parents wanted prior to making a decision.

A few Somali, Black Christian and Black Caribbean mothers were concerned that their biology made their child more at risk of disease or immunisation side effects. Somali parents were principally cautious of MMR vaccine for this reason, discussing concern about side effects (developmental issues and autism). One parent considered Somali boys to be particularly at risk. Some Asian Christian mothers expressed anxiety that immunisation research is not ethnically heterogeneous.

Everybody's body inside is different. You speak to white people you speak to Asian you speak to black, the color of our skin is all different, but is the inside of our bodies different? How are we inside? Do we all have the same mechanisms? Marlow, participant comment.

Participants described the information they received about immunisation as limited because it did not acknowledge these differences. Many articles suggested that parents wanted personalised information. African Caribbean and Somali mothers, in particular, wanted information that addressed the concerns of their specific community and that was supported by general practitioners (GPs) from their community. A problem raised by several women regarding meningitis immunisation was that leaflets could not depict a meningitis rash on black skin.

## Discussion

This systematic review identified that concepts related to ethnicity and perceptions of ethnic differences affect immunisation decision-making among parents from BAME backgrounds, living in the UK. Factors related to ethnicity, such as religion, upbringing and migration and language, affected parents' perceived importance of immunisations, whether immunisations were permitted or culturally acceptable and their understanding of immunisation/the immunisation schedule, which may have facilitated or inhibited immunisation. Beliefs about ethnic differences resulted in some parents being concerned about biological differences in risk of disease or immunisation side effects or that the UK environment was riskier than their ‘home’ environment. Beliefs about ethnic differences caused parents to demand tailored information.

Ethnicity is a complex construct and its different facets and associated factors should be considered when seeking to understand the immunisation decisions of parents from BAME backgrounds. Most quantitative immunisation research categorises participants into a limited number of ethnic groups (‘White’, ‘Black’, ‘Asian’ or ‘other’; although ethnicity-focused research often measures ethnicity in a more nuanced way) and few qualitative studies in this review described their sample in terms of the different facets of ethnicity. Although religion is associated with ethnicity, it may be considered independently. Some of the perspectives detailed in this review of parents from BAME backgrounds may equally be expressed by religious non-BAME parents and this review does not allow us to assess the prevalence of such perspectives.

Some parents' beliefs about ethnic differences between them and the majority population affected their decision-making. Similar perceptions about biological differences at the individual-level have been found among parents regardless of ethnicity (Forster *et al*, in preparation). Vaccination information currently provided by programme co-ordinators across the world, typically seeks to be ethnically inclusive or ethnically neutral.^31–33^ However, it appears that some parents would prefer to receive ethnicity-specific information that addresses the particular concerns of their ethnic group. Computer-tailored health communications are effective at changing individuals' behaviour (although effect sizes are small), and are more effective than targeted or generic messages.^34^ However, tailoring requires recipients to have had an individualised assessment before receiving the communication, which is unfeasible on a population-level for immunisation. There is evidence that targeted health communications (those developed with a particular subpopulation in mind, a more simplified version of tailored communications) are as effective as tailored communications when the information is a ‘good fit’ with the recipient.^35^ Where appropriate and feasible, vaccination information should be targeted to address parents' beliefs that biological ethnic differences increase risk of vaccine side effects. Intensive service delivery interventions, particularly systematic call–recall systems, have also improved vaccine uptake in areas with an ethnically diverse population.^36^ However, lower uptake among some ethnic groups remained in this study and interventions targeted at difficult to reach groups may be required to improve coverage, although will require significant development and trialling prior to implementation.

The review was limited by the range of ethnic labels used by the primary authors. It was often not clear whether participants' ethnicity was self-reported or assigned. The lack of diversity in individuals from different ethnic backgrounds means that we cannot draw conclusions about a particular ethnic group. We also cannot draw conclusions about whether parents with particular views are less likely to immunise their child, or choose certain vaccines but not others. While this review provides a better understanding of the experiences of some individuals from some BAME groups, it is important to note that individuals with the same ethnic background are not homogenous and do not share the same experiences, which can impact on health and socioeconomic position.^37–39^ Within religions, scriptural passages that could apply to vaccines are not interpreted uniformly.^40^ For these reasons, our findings are unlikely to be representative of all individuals from all BAME groups. The review identified many issues unrelated to ethnicity that have not been discussed here and are commonly expressed by parents in general. In interpreting these findings, one must consider ethnicity-specific issues alongside the common concerns about immunisation. Only eight articles were included in this review, suggesting that there is a need for more primary research. Most articles focused on MMR immunisation, which limits the extent to which we can generalise our findings to other immunisations and additional research should consider focusing on other vaccines in the childhood immunisation programme.

Immunisation decision-making among parents from BAME backgrounds is affected by religion, language, and upbringing and migration, as well as beliefs about ethnic differences. While there is a need for further primary research in this area, immunisation programme coordinators may be able to increase uptake of immunisations if their immunisation information is targeted to address ethnicity-specific concerns about immunisations alongside those that are expressed by parents in general. Interventions to increase uptake of immunisation, targeted at parents from BAME backgrounds, should be developed to take a multifaceted approach to ethnicity.

**What is already known on this subject**

- Immunisation is a vital public health intervention that has reduced morbidity and mortality from disease.
- Although uptake of immunisations in the UK is good in general, uptake among individuals from some Black and Asian Minority Ethnic (BAME) backgrounds is lower for some immunisations.
- Previous reviews have explored the factors that affect parents' decisions to immunise a child but have not considered whether ethnicity-specific factors influence the decisions of parents from BAME backgrounds.

**What this study adds**

- Our systematic review of qualitative research identified that factors related to ethnicity, namely religion, upbringing, migration and language, affect parents' immunisation decisions.
- We also found that some parents' beliefs about biological differences between individuals from BAME backgrounds and the majority population in the UK affected their decisions, as well as the information they wanted to receive prior to making a decision.
- Factors related to ethnicity should be considered when seeking to understand the immunisation decisions of parents from BAME backgrounds. Where possible, vaccination information should be targeted to address parents' ethnicity-specific concerns.

## Footnotes

i Papers including individuals identifying as Irish, Gypsy Traveller or any other White background were eligible for inclusion.

ii The total is not equal to 209 as one article reported using a mixed BAME sample but did not provide a breakdown of participants’ ethnicities.
