# Supplementary material for: Ethnicity-specific factors influencing childhood immunisation decisions among Black and Asian Minority Ethnic groups in the UK: a systematic review of qualitative research
Source: J Epidemiol Community Health. 2017 May 12;71(6):544–9. doi: 10.1136/jech-2016-207366 (PMC5484038; doi:10.1136/jech-2016-207366)
Supplement: supplementary material [file jech-2016-207366supp001.pdf]

Supplementary Material

Supplementary Material – Search terms by database

	PsycINFO	MEDLINE	Embase	Social Policy and Practice	CINAHL plus	Web of science
**Vaccination**	exp Immunization/<br>"vaccination*".ab,ti.<br>"immuni?ation*".ab,ti.	exp Immunization/<br>"vaccination*".ab,ti.<br>"immuni?ation*".ab,ti.	exp Immunization/<br>"vaccination*".ab,ti.<br>"immuni?ation*".ab,ti.	exp Immunization/<br>"vaccination*".ab,ti.<br>"immuni?ation*".ab,ti.	Exp Immunization/<br>“Immuni?ation*”<br>“Vaccination*”	“Immuni?ation*”<br>“Vaccination*”
**Qualitative**	exp Qualitative Research/<br>exp Interviews/<br>exp Observation Methods/<br>"interview*".ab,ti.<br>"focus group*".ab,ti.<br>"ethnogr*".ab,ti.<br>exp Ethnography/<br>“thematic analysis”.ab,ti.<br>“grounded theory”.ab,ti.<br>“interpretative phenomenological analysis”.ab,ti.<br>“content analysis”.ab,ti.<br>“framework analysis”.ab,ti.	exp Qualitative Research/<br>exp Interviews/<br>exp Observation Methods/<br>"interview*".ab,ti.<br>"focus group*".ab,ti.<br>"ethnogr*".ab,ti.<br>exp Ethnography/<br>“thematic analysis”.ab,ti.<br>“grounded theory”.ab,ti.<br>“interpretative phenomenological analysis”.ab,ti.<br>“content analysis”.ab,ti.<br>“framework analysis”.ab,ti.	exp Qualitative Research/<br>exp Interviews/<br>exp Observation Methods/<br>"interview*".ab,ti.<br>"focus group*".ab,ti.<br>"ethnogr*".ab,ti.<br>exp Ethnography/<br>“thematic analysis”.ab,ti.<br>“grounded theory”.ab,ti.<br>“interpretative phenomenological analysis”.ab,ti.<br>“content analysis”.ab,ti.<br>“framework analysis”.ab,ti.	exp Qualitative Research/<br>exp Interviews/<br>exp Observation Methods/<br>"interview*".ab,ti.<br>"focus group*".ab,ti.<br>"ethnogr*".ab,ti.<br>exp Ethnography/<br>“thematic analysis”.ab,ti.<br>“grounded theory”.ab,ti.<br>“interpretative phenomenological analysis”.ab,ti.<br>“content analysis”.ab,ti.<br>“framework analysis”.ab,ti.	exp Qualitative Studies/<br>exp Anthropology, Cultural/<br>exp Ethnographic Research/<br>exp Interviews/<br>exp Semi-structured Interview/<br>exp Structured Interview/<br>exp Focus Group/<br>exp Nonexperimental Studies/<br>exp Content Analysis/<br>exp Thematic Analysis/<br>exp Grounded Theory/<br>Qualitative*<br>Anthropology*<br>Ethnog*<br>Interview*<br>“Focus group”*<br>Observation*<br>“Content analysis”<br>“Thematic analysis”<br>“Grounded theory”	Qualitative*<br>Interview*<br>“Focus group”*<br>Observat*<br>Ethnog*Anthrop*<br>“Content analysis”<br>“Framework analysis”<br>“Interpretative phenomenological analysis”<br>“Grounded theory”
**United Kingdom**	"United Kingdom".ab,ti.<br>"Great Britain".ab,ti.<br>England.ab,ti.<br>Scotland.ab,ti.<br>Wales.ab,ti.<br>"Northern Ireland".ab,ti.	"United Kingdom".ab,ti.<br>"Great Britain".ab,ti.<br>England.ab,ti.<br>Scotland.ab,ti.<br>Wales.ab,ti.<br>"Northern Ireland".ab,ti	"United Kingdom".ab,ti.<br>"Great Britain".ab,ti.<br>England.ab,ti.<br>Scotland.ab,ti.<br>Wales.ab,ti.<br>"Northern Ireland".ab,ti	"United Kingdom".ab,ti.<br>"Great Britain".ab,ti.<br>England.ab,ti.<br>Scotland.ab,ti.<br>Wales.ab,ti.<br>"Northern Ireland".ab,ti	United Kingdom/<br>England/<br>Great Britain/<br>Scotland/<br>Wales/<br>Northern Ireland/<br>“United Kingdom”<br>“Great Britain”<br>“England”<br>“Northern Ireland”<br>“Wales”<br>“Scotland”	“United Kingdom”<br>“Great Britain”<br>“England”<br>“Northern Ireland”<br>“Wales”<br>“Scotland”

Supplementary Material – Summary of findings that did not relate to ethnicity.

Issues unrelated to ethnicity were raised in the data and are not discussed in the results. These issues were those commonly expressed by parents in general with regard to immunisation decision-making (including parents being happy to comply with the recommended immunisation schedule, differing perceptions of responsibility to vaccinate, influence of personal experience and of others' advice, concerns about vaccines, perceptions that children's vulnerability to disease or vaccine side-effects is individualised and practical issues making vaccination difficult).

Supplementary Material - Characteristics of included articles

Lead author	Aim	Population of interest	Number of participants from BAME backgrounds; Gender	Ethnicity	Data collection period	Study Design	Analysis	Immunisation of interest	Risk of bias
Brown (2012)	To explore parents' MMR decision-making	Mothers planning to accept, postpone or decline the first MMR dose for their 11-36 month old children	16; Female	6; White non-British, 2; Black African, 2; Black British, 1; Chinese, 2 Asian British, 2; Asian, 1; Mixed British/African<br><br>(Total sample size = 24) [a]	June 2008 to March 2009	Semi-structured interviews	Modified grounded theory	MMR	Low
Condon (2002)	To explore the attitudes of ethnic minority parents to preschool immunisations, particularly first MMR immunisation	Mothers of children aged 16 months to 3 years of Pakistani, Somali and Afro-Caribbean ethnicity	21; Female	11; Pakistani, 5; Somali, 5; Afro-Caribbean<br><br>Languages spoken included English, Somali and Punjabi/Urdu<br><br>Some participants were born in the UK, some were not	November 2000 to March 2001	Semi-structured interviews and focus groups	Thematic analysis	Childhood immunisation in general	Low
Hill (2013)	To ascertain factors influencing parental immunisation decision making	Parents of children who have received the MMR immunisation	3; Female	1; Ghanaian, 1; Turkish, 1; British Black<br><br>Language spoken was English<br><br>(Total sample size = 5) [a]	July 2010	Semi-structured interviews	Modified grounded theory	MMR	Low

Johnson (2014)	To explore mothers' engagement with advice around the combined MMR immunisation	Mothers of children aged 12-18 months	1; Female	1; Eastern European<br><br>(Total sample size = 5) [a]	2011	Focus groups	Thematic analysis	MMR	Low
Marlow (2009)	To explore attitudes to HPV immunisation among Black and Asian mothers living in Britain	Black and Asian mothers living in Britain	20; Female	*Described by ethnic group, religion and migration status:*<br><br>6; Black Caribbean, 4; Black African, 3; Asian Indian, 3; Asian Pakistani, 1; Asian Bangladeshi, 3; Asian other<br><br>7; Black Christian, 1; Asian Christian, 4; Asian Hindu, 1; Black Muslim, 5; Asian Muslim, 2; Black no religion<br><br>12; Born in UK, 8; Not born in UK	April 2008 to August 2008	Interviews	Framework analysis	HPV	Low
Mixer (2007)	To explore ethnic differences in knowledge, attitudes and behaviour related to immunisation	Mothers from various ethnic backgrounds	24; Female	12; Afro-Caribbean, 12; Asian<br><br>Languages spoken included English and Gujarati<br><br>(Total sample size = 37) [a]	Not described	Focus groups	Thematic analysis	MMR	Low

Supplementary Material - Characteristics of included articles *(continued)*

Petts (2004)	To describe the information strategies that parents use to make sense of health risk issues	Parents of children with various MMR immunisation status'	64; Gender unknown [b]	At least 28 Asian Muslim mothers	February 2002 to July 2002	Focus groups	Analytic deduction	MMR	Low
Tomlinson (2013)	To explore the health beliefs of Somali women resident in the UK	Somali women resident in the UK with one child <5 years of age	23; Female	23; Somali<br><br>Islam<br><br>Languages spoken included English and Somali<br>23; Not born in UK	February 2012 to April 2012	Semi-structured interviews	Thematic analysis	Childhood immunisation in general	Low

[a] We included articles that reported data from White British participants but these data were not included in the review. The 'total sample size' denotes the total study sample size where participants who were not from a BAME background were included in the article.

[b] The 'total number of participants' column refers to the total number of participants in the article who were from a BAME background. Gender was not broken down by ethnicity in two articles which also included White British participants. For this reason, for these articles, we cannot report the gender of participants from BAME backgrounds.

Supplementary Material – References of articles included in the review

Brown KF, Long SJ, Ramsay M, Hudson MJ, Green J, Vincent CA, et al. U.K. parents' decision-making about measles-mumps-rubella (MMR) vaccine 10 years after the MMR-autism controversy: a qualitative analysis. Vaccine 2012;30(10):1855-1864.

Condon L. Maternal attitudes to preschool immunisations among ethnic minority groups. Health Education Journal 2002;61(2):180-189.

Hill MC, Cox CL. Influencing factors in MMR immunisation decision making. British Journal of Nursing 2013;22(15):893-898.

Johnson S, Capdevila R. 'That's just what's expected of you … so you do it': Mothers discussions around choice and the MMR vaccination. Psychology & Health 2014;29(8):861-876.

Marlow LA, Wardle J, Waller J. Attitudes to HPV vaccination among ethnic minority mothers in the UK: an exploratory qualitative study. Human Vaccines 2009;5(2):105-110.

Mixer RE, Jamrozik K, Newsom D. Ethnicity as a correlate of the uptake of the first dose of mumps, measles and rubella vaccine. Journal of Epidemiology & Community Health 2007;61(9):797-801.

Petts J, Niemeyer S. Health risk communication and amplification: learning from the MMR vaccination controversy. Health, Risk & Society 2004;6(1):7-23.

Tomlinson N, Redwood S. Health beliefs about preschool immunisations: an exploration of the views of Somali women resident in the UK. Diversity & Equality in Health & Care 2013;10(2):101-113.

Supplementary Material – Inclusion and exclusion criteria

Types of studies

Primary research studies meeting the following criteria:

- reporting qualitative analysis of textual data (collected using focus groups, interviews, participant observation, free-text questionnaire responses);
- and indexed at any time in online databases and published in peer reviewed journals in English.

We excluded dissertation abstracts, book chapters, review articles and commentaries.

Types of participants

Parents or caregivers of children/adolescents living in the United Kingdom. Participants must have been making decisions about vaccinating a child (under 18 years old). Participants must be from a Black or Asian Minority Ethnic group (defined as being not White English/Welsh/Scottish/Northern Irish/British).
